# Preparing students for lifelong learning by means of metacognition

**DOI:** 10.3205/zma001347

**Published:** 2020-09-15

**Authors:** Marjo Wijnen-Meijer

**Affiliations:** 1 Technical University of Munich, TUM School of Medicine, TUM Medical Education Center (TUM MEC), Munich, Germany

## Editorial

Discussions about the content and competences of a (medical) study programme mainly focus on what a person needs to learn in order to be able to work or to do a residency programme immediately after graduation. But actually, one should also consider what the graduates need for the (longer) period after that. As Hippocrates said: “The life so short, the craft so long to learn” [1]. Because the clinical environment keeps changing, medical trainees need to become adaptive experts. Adaptive experts are able to perform routine tasks efficiently and, at the same time to find solutions to new problems and to adapt their way of working to new circumstances [2], [3]. 

That means that learning does not stop after finishing medical school and it is therefore important that students are getting prepared to do that in the rest of their lives. I think David Sackett explained very well the importance of self-directed learning in the article that is called “Thoughts for new medical students at a new medical school”: “Half of what you’ll learn at medical school will be shown to be either wrong or out of date within 5 years of graduation; the trouble is that nobody can tell you which half, so the important thing to learn is how to learn on your own.” [4]. 

“Learning on your own” is often referred so as self-directed or self-regulated learning. In the literature, there are many definitions and descriptions of this term, for instance “As the preparedness of a student to engage in learning activities defined by himself rather than a teacher”, “Organizing teaching and learning so that learning is within the learners’ control” or “an independent pursuit that involves a philosophy of personal autonomy and self-management” [5]. A number of authors describe self-regulated learning as a process in which different phases follow each other cyclically. A well-known model is that of Zimmerman who distinguishes three phases: performance phase, self-reflection phase and forethought phase [6]. Sanders & Cleary [7] provide a more comprehensive description of self-regulation: “the cyclical control of academic and clinical performance through several key processes that include goal-directed behaviour, use of specific strategies to attain goals, and the adaptation and modification to behaviours or strategies to optimise learning and performance.”

What everyone agrees on: self-regulated learning requires metacognition, which is, in short, “thinking about one’s thinking” [8]. It means that people can assess their own capabilities and determine the right strategies to acquire the right knowledge or to solve a specific problem. 

In the literature three components of metacognition are distinguished [8], [9], [10]. The first component is metacognitive knowledge. This is also called metacognitive awareness and it means being able to make a correct assessment of what one knows and does not know. It also includes knowledge about and on problem-solving strategies. The second component concerns metacognitive skills (or metacognitive regulation). These are skills such as goalsetting, selecting accurate strategies, allocating resources and evaluating the result of the task performance. The third component, metacognitive experiences, are experiences that have something to do with the cognitive effort, for instance a feeling of confusion because the person does not fully understand something (yet) or an aha-experience.

In recent years there has been more research into the development of metacognition, and it turns out that this does not happen "by itself" [11]. Unfortunately, medical training pays too little explicit attention to metacognition and self-regulated learning [7], although metacognitive skills are learnable and teachable [12]. Like other skills, one can develop these skills by practicing them consciously on a regular basis.

In several articles described in this issue, didactic approaches are described that (indirectly) contribute to the development of metacognition of students.

An important factor for metacognitive knowledge is that someone has insight into his or her level of knowledge, and into the way this knowledge develops. A progress test, as described in the article by Zupanic et al. [13], can be very useful.In addtion, if students regularly receive concrete feedback about their knowledge and skills, this contributes to the development of their metacognition. The article of Thrien et al. [14] describes examples of giving feedback in practice.

 Examination anxiety can have a major impact on exam results and on the way students prepare for exams. The risk is that students have bad experiences with exams over and over again, which increases the influence of examination anxiety. If students understand the background of their fear, they can better adapt their learning strategies accordingly. The development of an instrument to measure examination anxiety, as described in the article by Stefan et al. [15], can therefore make an important contribution to students' metacognitive skills. An important aspect of metacognition, is the ability to (self) reflexion. Scheide et al. [16] describe in their article how a specific seminar contributes to this. 

If students themselves fulfil the role of (peer) teacher, this can also have a positive effect on their metacognitive knowledge and skills. By explaining knowledge to others, students gain better insight into their own level of knowledge. In addition, when they learn to understand didactic principles, they are able to choose more effective learning strategies [17]. Therefore, the format described in the article by Koenenmann et al [18], in which peer-teachers supervise clinical case discussions, is a valuable didactic concept. Finally, it is also important that teachers and supervisors understand how to ensure that students gain valuable learning experiences. In this context, didactic qualifications, as described in the article by Böhme et al. [19], make an important contribution to the development of the metacognition of students, and of course the teachers themselves.

Let us, as educators, take responsibility and prepare students for lifelong learning by regularly paying attention to metacognition.

## Competing interests

The author declares that she has no competing interests.
